# Potential Protective Effect of Hepatitis B Immunity Against Diabetes Mellitus: A Retrospective Propensity-Matched Cohort Study

**DOI:** 10.3390/diagnostics15131610

**Published:** 2025-06-25

**Authors:** Nhu Quynh Phan, Shih-Jung Lin, Ngoc Hoang Le, Van Thuan Nguyen, Tan Ha Mai, Jin-Hua Chen, Min-Huei Hsu, Dinh Khanh Hoang, Phung Manh Thang, Ya-Li Huang, Chiehfeng Chen

**Affiliations:** 1 International Ph.D. Program in Medicine, College of Medicine, Taipei Medical University, Taipei 11031, Taiwand142113001@tmu.edu.tw (D.K.H.); 2 Department of Infection Control, Cho Ray Hospital, Ho Chi Minh City 700000, Vietnam; 3 School of Medicine, College of Medicine, Taipei Medical University, Taipei 11031, Taiwan; 4 Graduate Institute of Biomedical Materials and Tissue Engineering, College of Biomedical Engineering, Taipei Medical University, Taipei 11031, Taiwan; 5 Department of Computer Science and Information Engineering, National Taiwan University, Taipei 10617, Taiwan; 6 Graduate Institute of Data Science, College of Management, Taipei Medical University, Taipei 11031, Taiwan; 7 Research Center of Biostatistics, College of Management, Taipei Medical University, Taipei 11031, Taiwan; 8 Biostatistics Center, Wan Fang Hospital, Taipei Medical University, Taipei 11695, Taiwan; 9 Department of Histopathology, Hai Phong University of Medicine and Pharmacy, Hai Phong 180000, Vietnam; 10 Department of Public Health, School of Medicine, College of Medicine, Taipei Medical University, Taipei 11031, Taiwan; 11 Cochrane Taiwan, Taipei Medical University, Taipei 11031, Taiwan; 12 Evidence-Based Medicine Center, Wan Fang Hospital, Taipei Medical University, Taipei 11695, Taiwan; 13 Division of Plastic Surgery, Department of Surgery, Wan Fang Hospital, Taipei Medical University, Taipei 11695, Taiwan

**Keywords:** hepatitis B, vaccination, immunity, diabetes mellitus, prevention

## Abstract

**Background**: Hepatitis B virus (HBV) infection affects glucose metabolism and increases diabetes risk; HBV vaccination may reduce this risk. The role of HBV immunity in diabetes prevention among individuals without HBV infection is underexplored. This study aims to evaluate whether HBV immunity reduces diabetes risk in individuals without HBV infection. **Methods**: This retrospective cohort study used de-identified electronic medical records from TriNetX. Adults with hepatitis B surface antibody (HBsAb) results without a history of HBV infection or diabetes were identified. Diabetes was defined on the basis of a diabetes diagnosis, diabetes medication use, or glycated hemoglobin ≥ 6.5%. Propensity score matching was conducted to balance demographics and comorbidities between groups. **Results**: The HBV-immunized group had a 15% lower diabetes risk than the HBV-unimmunized group (HR: 0.85 [0.84–0.87]). A dose–response effect was observed, with higher HBsAb levels showing a greater reduction in the risk of diabetes. HBsAb levels of ≥100 and ≥1000 mIU/mL were associated with 19% (HR: 0.81 [0.80–0.83]) and 43% (HR: 0.57 [0.54–0.60]) reductions in diabetes risk, respectively, compared with HBsAb < 10 mIU/mL. The reduced risk of diabetes was associated with age. Immunized individuals aged 18 to 44 years, 45 to 64 years, and ≥65 years had 20% (HR: 0.80 [0.78–0.82]), 11% (HR: 0.89 [0.87–0.92]), and 12% (HR: 0.88 [0.84–0.91]) lower diabetes risks, respectively, compared with unimmunized individuals. **Conclusions:** HBV immunity may be associated with a reduced risk of diabetes, suggesting broader HBV vaccination as a dual-benefit strategy for the prevention of hepatitis B and diabetes, especially in regions with a high prevalence of both diseases.

## 1. Introduction

Diabetes mellitus is a major and growing global health concern characterized by persistent hyperglycemia and serious complications such as cardiovascular disease, kidney failure, and blindness [1]. In 2024, an estimated 588.7 million people were living with diabetes, and this number is projected to rise by 45% to 852.5 million by 2050 if current trends continue. Also in 2024, diabetes caused over 3.4 million deaths, accounting for 9.3% of all global mortality. The economic burden is also substantial, with global health expenditure related to diabetes surpassing USD 1 trillion, equivalent to 12% of total healthcare spending [2]. Given the escalating burden of diabetes globally, effective prevention strategies are urgently required to halt the progression of diabetes and alleviate its substantial health and economic burdens.

In addition to the regulatory role of the pancreas, the liver is also essential for maintaining glucose homeostasis. Specifically, it maintains plasma glucose levels through gluconeogenesis and glycogenolysis during fasting states. Conversely, after meals, the liver lowers blood glucose by converting excess glucose into glycogen via glycogenesis [3]. Previous studies have indicated that hepatitis B virus (HBV) infection may disrupt this balance by promoting gluconeogenesis and altering key metabolic pathways in hepatocytes [4]. Specifically, HBV X protein (HBx) may promote gluconeogenesis by upregulating the expression of genes for key gluconeogenic enzymes, leading to increased glucose production and the development of hyperglycemia and impaired glucose tolerance [5]. Several studies have discovered a higher prevalence of diabetes in patients with HBV infection compared with those without HBV infection [6,7]. A 12-year follow-up cohort study provided novel evidence of an association between HBV infection and the risk of diabetes. The results reveal that diabetes may be an additional metabolic complication of HBV infection [8]. Consequently, the effective prevention of HBV infection may have significant implications for diabetes prevention strategies.

HBV vaccination is an effective method for activating the immune system and preventing HBV infection [9], which in turn may reduce the risk of diabetes. Hepatitis B surface antibody (HBsAb), which indicates the presence of immunity against HBV, can be produced either after recovery from HBV infection or following HBV vaccination [10]. Huang et al. (2015) indicated that vaccination against HBV not only protects against HBV infection but may also play a role in reducing the risk of diabetes by enhancing immune responses; successful HBV vaccination could decrease the risk of diabetes by 33% [11]. This result suggests that HBV vaccination prevents diabetes, especially among at-risk patients with HBV.

Although the significant association between HBV infection and diabetes risk has been welldocumented in several observational studies [12,13], the potential protective effect of HBV immunity against diabetes has not been thoroughly examined. Preliminary evidence suggests that successful HBV vaccination reduces the risk of diabetes [11], potentially by preventing infection with the virus and thereby preventing liver damage and cirrhosis, which are the complications of HBV infection that affect glucose metabolism. However, whether the protective effects of HBV vaccine-induced immunity against diabetes are independent of infection prevention remains unclear. Recent theories have proposed that vaccination might decrease the risk of chronic diseases, including diabetes, by both preventing infection and enhancing immune regulation [14,15]. To address this research gap, our study investigated whether HBV immunity, as measured by HBsAb titers, is independently associated with a reduced risk of diabetes mellitus in individuals without HBV infection.

## 2. Materials and Methods

### 2.1. Database

This retrospective cohort study was conducted using data from TriNetX (TriNetX Inc., Cambridge, MA, USA). TriNetX is a global platform that provides access to a comprehensive network of de-identified electronic medical records (EMRs) that contain data on diagnoses, procedures, medications, laboratory test results, and genomic information that can be used for biomedical and clinical research [16]. A real-time analysis of the Global Collaborative Network on the TriNetX platform was conducted in October 2024. This study did not involve any intervention or interaction with human subjects, and data were de-identified following the standards outlined in Section §164.514(a) of the Health Insurance Portability and Accountability Act (HIPAA) Privacy Rule. The de-identification process was confirmed by a formal determination from a qualified expert as specified in Section §164.514(b)(1) of the HIPAA Privacy Rule, which was last updated in December 2020. Given the de-identified nature of the data, this study was granted exemption from informed consent requirements.

### 2.2. Study Participants and Cohorts

This study included adults (aged ≥ 18 years) with medical records mentioning HBsAb serology test results between 1 January 2005 and 31 December 2023. Patients with a history of HBV infection, as indicated by a HBV diagnosis, hepatitis B surface antigen (HBsAg) positivity, or hepatitis B core antibody (HBcAb) positivity [10], were excluded from this study (Supplementary Table S6). The index date was the date of the HBsAb serology test.

The study participants were divided into two cohorts on the basis of HBV immunization status. The HBV-immunized group comprised individuals who were HBsAb-positive (≥10 mIU/mL), and the HBV-unimmunized group comprised individuals who were HBsAb-negative (<10 mIU/mL). Because patients with a history of HBV infection were excluded from our study, the positive HBsAb status in the immunized group likely resulted from vaccination. By contrast, in the control group, HBsAb negativity likely resulted from non-vaccination or vaccination without subsequent immunity development [10].

**Figure 1:**
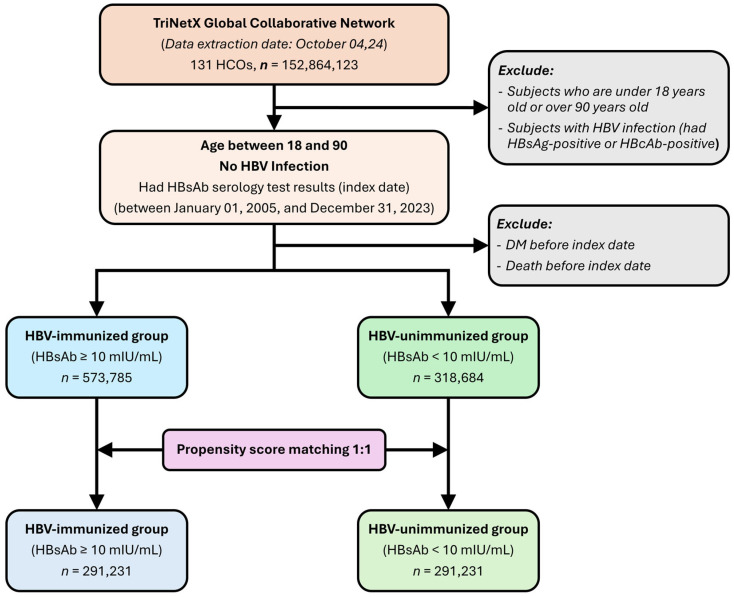
Flowchart of participant selection.

Patients who developed diabetes before the index date, as indicated by a diabetes diagnosis (International Classification of Diseases, Tenth Revision, Clinical Modification [ICD-10-CM] codes E08–E13); the use of antihyperglycemic drugs; or glycated hemoglobin (HbA1c) levels ≥ 6.5%, were excluded from both groups (Figure 1).

### 2.3. Outcomes

In this study, the outcome was new-onset diabetes, which was identified using (1) ICD-10-CM codes E08–E13, (2) the use of antihyperglycemic drugs, and (3) HbA1c level ≥ 6.5%. This study examined a composite outcome including any of (1), (2), or (3).

Within each group, patients were followed up for the occurrence of outcomes from the first day to 15 years after the index date.

### 2.4. Statistical Analysis

To minimize exposure selection bias, the study and comparison groups were matched using propensity score matching (PSM) at a 1:1 ratio to balance the distribution of covariates between the groups, including age at the index date, sex, ethnicity, BMI, HbA1c, socioeconomic and psychosocial circumstances, lifestyle factors (including tobacco use, nicotine dependence, and alcohol-related disorder), and comorbidities. Baseline comorbidities were hypertensive diseases, hyperlipidemia, heart failure, ischemic heart diseases, chronic kidney disease, overweight and obesity, and liver diseases (Supplementary Table S6). Baseline comorbidities were identified on the basis of diagnoses in EMRs before the index date. On the TriNetX platform, logistic regression analysis was implemented using user-defined covariate matrices to calculate propensity scores for each subject. These scores were then employed to pair patients using the greedy nearest-neighbor method, with a caliper size set to 0.1 of the pooled standard deviation. To prevent the bias often associated with nearest-neighbor matching, the order of the data rows was shuffled using the TriNetX platform. The balance of covariates was evaluated on the basis of standardized mean difference (SMD) values. SMD ≤ 0.1 indicated well-balanced covariates between the groups. Baseline characteristics are reported using descriptive statistics, namely the mean and standard deviation for continuous variables and the count and proportion for categorical variables.

Kaplan–Meier analysis was conducted to determine the cumulative incidence of outcomes between the two groups. The log-rank test was used to assess the differences in the probability of outcomes between the groups. Cox proportional hazards regression models were used to calculate hazard ratios (HRs) and 95% confidence intervals (CIs) by running a suite of tests with R’s Survival package v3.2-3 (R Foundation for Statistical Computing, Vienna, Austria). The results were validated by comparing the numbers with output from SAS version 9.4 (SAS Institute Inc., Cary, NC, USA).

To evaluate the dose–response relationship between hepatitis B surface antibody (HBsAb) levels and the risk of diabetes, we conducted an analysis using three separate Cox proportional hazards regression models. HBsAb concentrations were categorized into four groups based on clinically relevant thresholds: <10 mIU/mL (reference), ≥10 mIU/mL, ≥100 mIU/mL, and ≥1000 mIU/mL. The threshold of 10 mIU/mL is widely accepted as the minimum protective level against hepatitis B virus (HBV) infection, endorsed by professional societies such as the Infectious Diseases Society of America (IDSA) and the American Society for Microbiology (ASM) [17]. A level of ≥100 mIU/mL is recommended for high-risk populations, including healthcare workers and individuals with isolated anti-HBc or those living with HIV, to ensure a more sustained immune response [18,19]. Although rarely applied in routine clinical practice, a threshold of ≥1000 mIU/mL has been used in research settings to represent robust and long-lasting immunity, particularly among immunocompromised populations [20].

We performed stratified analyses to assess whether the risk of diabetes mellitus differed by region, sex, and age. For the analysis stratified by region, we used data from four regional collaborative networks on the TriNetX platform, namely the United States; Latin America; Europe, Middle East, and Africa; and Asia-Pacific networks.

To evaluate the robustness of the association between HBV immunity and diabetes risk, we conducted a sensitivity analysis using alternative HBsAb thresholds. Specifically, we compared the risk of diabetes among individuals with HBsAb levels ≥ 100 mIU/mL versus those with levels < 100 mIU/mL, and, separately, individuals with HBsAb levels ≥ 1000 mIU/mL versus those with levels < 1000 mIU/mL (Supplementary Table S5).

All statistical analyses were conducted using the browser-based, real-time analytics feature on the TriNetX Live platform. A two-sided *p* < 0.05 was considered statistically significant. Figures were generated using R version 4.4.0.

## 3. Results

### 3.1. Baseline Characteristics

The Global Collaborative Network includes data from more than 152 million people from 131 healthcare organizations (HCOs). After the application of the inclusion and exclusion criteria, our retrospective cohort study included 573,785 individuals in the HBV-immunized group (HBsAb ≥ 10 mIU/mL) and 318,684 individuals in the HBV-unimmunized group (HBsAb < 10 mIU/mL). Before PSM, differences in age and other demographics were observed between the two groups. After PSM, demographics were well balanced between the groups. Each group contained 291,231 individuals (Figure 1), with a mean age of 42.1 ± 16.1 years. Approximately 56% of both groups were women, and nearly 60% in each group were White individuals. Comorbidities, including hypertension, hyperlipidemia, heart failure, ischemic heart diseases, chronic kidney disease, overweight/obesity, and liver diseases, were also well balanced after matching (Supplementary Table S1).

### 3.2. The HBV-Immunized Group Had a Lower Risk of New-Onset Diabetes

**Figure 2:**
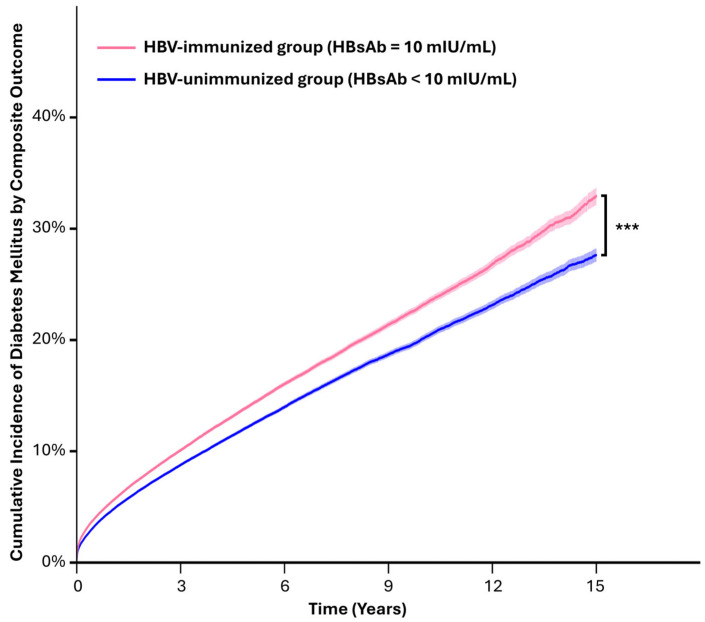
Kaplan–Meier curves of cumulative incidence of diabetes mellitus by composite outcome. *** *p* log-rank test < 0.001.

In our analysis, the HBV-immunized group exhibited a significantly lower risk of new-onset diabetes than the HBV-unimmunized group. Kaplan–Meier curves were employed to visualize the cumulative incidence of diabetes mellitus between the groups based on the examination of the composite outcome over time. The curves indicated a consistently lower probability of diabetes onset in the HBV-immunized group than in the HBV-unimmunized group. Significant differences were noted between the groups, as confirmed by the *p* < 0.001 in the log-rank test (Figure 2).

These findings were confirmed by Cox regression models. As detailed in Table 1, compared with the HBV-unimmunized group, the HBV-immunized group exhibited a significant reduction in the risk of diabetes-related outcomes. Specifically, the risk of new-onset diabetes mellitus, as determined by the composite outcome, was reduced by 15% (HR: 0.85, 95% CI: 0.84–0.87). Moreover, the probability of receiving a diabetes diagnosis based on *ICD-10-CM* codes in EMRs was 11% lower in the HBV-immunized group (HR: 0.89, 95% CI: 0.87–0.91). In the analysis, the risk of using antihyperglycemic drugs reduced by 16% in HBV-immunized individuals (HR: 0.84, 95% CI: 0.83–0.86). In addition, in the HBV-immunized group, the likelihood of elevated HbA1c levels (HbA1c ≥ 6.5%) substantially decreased by 25% (HR: 0.75, 95% CI: 0.72–0.77).

### 3.3. Dose–Response Analysis

**Figure 3:**
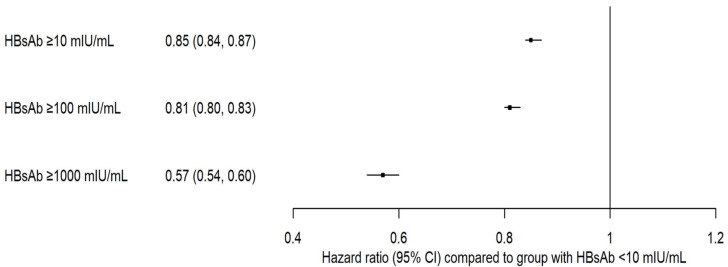
Dose–response effect of hepatitis B surface antibody levels and diabetes mellitus by composite outcome.

A dose–response relationship was observed between HBsAb levels and diabetes risk, with higher antibody concentrations being associated with a lower risk of diabetes. Individuals with HBsAb levels ≥ 10 mIU/mL exhibited a 15% reduction in the risk of the composite outcome compared with HBV-unimmunized individuals. Those with HBsAb levels ≥ 100 mIU/mL showed a 19% reduction in the risk (HR: 0.81; 95% CI: 0.80–0.83). Individuals with HBsAb levels ≥ 1000 mIU/mL exhibited the most marked reduction in the risk of incident diabetes, with a reduction of 43% (HR: 0.57, 95% CI: 0.54–0.60; Figure 3).

Dose–response effects were also consistently observed for the other diabetes-related outcomes, including diabetes mellitus diagnosis and the use of antihyperglycemic medications. However, for the outcome of elevated HbA1c levels (≥6.5%), although HRs decreased with increasing HBsAb levels, the CIs overlapped. Thus, the reduced risk of this specific outcome was less conclusive (Supplementary Figure S1).

### 3.4. Stratified Analysis

**Figure 4:**
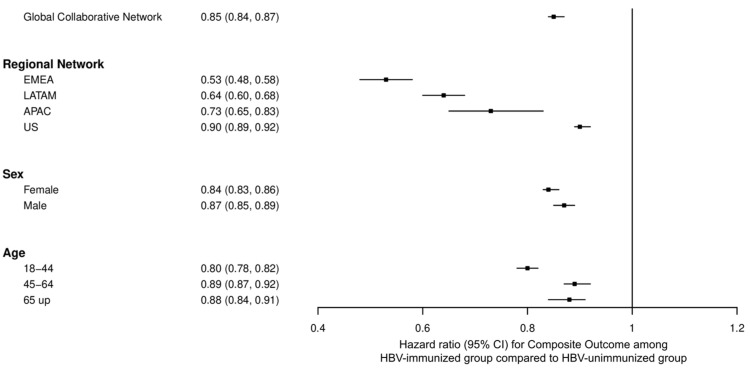
Risk of diabetes mellitus by composite outcome, stratified by region, age, and sex.

The association between HBV immunity and reduced risk of diabetes mellitus varied by region. It was most prominent in the Europe, Middle East, and Africa region, and least evident in the United States (Figure 4 and Supplementary Table S2). These findings indicate possible geographical differences in the immunological responses or epidemiological characteristics related to hepatitis B and diabetes.

Stratified analysis by sex revealed that the association between HBV immunity and reduced diabetes risk was similar in both men and women (Figure 4 and Supplementary Table S3). Additionally, in the analysis stratified by age, the results demonstrated a stronger association with lower diabetes risk in younger individuals (aged 18 to 44 years) than in middle-aged (aged 45 to 64 years) and older adults (aged ≥ 65 years). Specifically, among young adults, the risk of diabetes was 20% lower in the HBV-immunized group (HBsAb ≥ 10 mIU/mL) than in the HBV-unimmunized group (HBsAb < 10 mIU/mL). Moreover, the association between HBV immunity and lower diabetes risk persisted in middle-aged adults and older adults, although the strength of the association was reduced. Middle-aged adults and older adults had 11% and 12% lower risks, respectively (Figure 4 and Supplementary Table S4).

### 3.5. Sensitivity Analysis

Compared with individuals with HBsAb < 100 mIU/mL, those with HBsAb ≥ 100 mIU/mL had significantly lower risks of all diabetes-related outcomes. The association remained robust even when the threshold was increased to 1000 mIU/mL (Supplementary Table S5).

## 4. Discussion

To investigate the association between HBV immunity and diabetes risk, we conducted a retrospective cohort study using data from the Global Collaborative Network on the TriNetX platform. The main finding is that immunized (HBsAb-positive) individuals exhibited a 15% reduction in diabetes risk compared with unimmunized individuals (Table 1). Given that the study population included only those without HBV infection, the positive HBsAb status in the HBV-immunized group likely resulted from vaccination. This observation supported an association between HBV vaccine-induced immunity and diabetes prevention. A dose–response relationship was also observed, with higher antibody levels being associated with a lower risk of diabetes. Moreover, this association appeared stronger in younger individuals than in middle-aged and older adults.

Our main findings support the hypothesis that HBV immunity is associated with a reduced risk of diabetes, which is consistent with the results of previous studies. For example, Li et al. reported a lower prevalence of diabetes among HBsAb-positive retired women in China (OR: 0.579, 95% CI: 0.388–0.918) [21]. However, the study by Li et al. only excluded HBsAg-positive individuals at the time of data collection and did not consider HBcAb status; thus, the history of HBV infection was uncertain in some participants. Additionally, our study results are partially consistent with those of Huang et al., who used data from the NHANES database and reported a 33% reduction in diabetes risk among successfully vaccinated individuals [11]. Huang defined successfully vaccinated individuals as those who were HBsAb-positive, HBsAg-negative, and HBcAb-negative with a history of HBV vaccination. The control group included individuals with mixed immunity backgrounds, potentially including those previously infected with HBV. The inclusion of individuals with previous infection in the control group may contribute to higher diabetes risk due to liver damage from previous HBV infection, which may impair metabolic regulation and increase diabetes risk [8]. By focusing on individuals who have never been infected with HBV, the present study eliminated the influence of differing HBV infection rates between groups. Thus, this study reveals that the 15% reduction in diabetes risk is directly associated with HBV immunity. This finding suggests that HBV immunity may be associated with a lower risk of diabetes through mechanisms beyond the prevention of HBV infection, potentially involving unique metabolic pathways. Additionally, both the studies by Li et al. and Huang et al. had cross-sectional designs, limiting the strength of causal inference. Thus, from Li and Huang’s odds ratio results, besides concluding that HBV immunity reduces diabetes risk, it could also be inferred that individuals with diabetes may have a reduced ability to develop immunity after vaccination compared to those without diabetes. The present cohort study excluded individuals who had diabetes before the index date. Therefore, the strength of causal inference in this study is higher than that in cross-sectional studies, supporting a more reliable association between HBV immunity and reduced diabetes risk.

Some studies have provided inconsistent results for this association. Data from two large cross-sectional studies in Guangdong, China, revealed no association between serological HBsAb status and the presence of diabetes [22]. Similarly, a cohort study, also conducted in a Chinese population, initially found a lower diabetes risk in participants with HBsAg-negative/anti-HBs-positive/anti-HBc-negative status (implying immunity due to hepatitis B vaccination) than in those with HBsAg-negative/anti-HBs-negative/anti-HBc-negative status (indicating susceptibility to HBV) in an unadjusted model (HR: 0.89, 95% CI: 0.84–0.95, *p* = 0.0002). However, after adjustment for age, sex, and other covariates, this association became nonsignificant [23]. Therefore, more studies are warranted to confirm the relationship between HBV immunity and diabetes.

The results suggest that higher antibody concentrations are associated with stronger reductions in the risk of diabetes, as reflected in the composite outcome, diabetes mellitus diagnosis, and the use of any anti-hyperglycemic drugs. For the outcome of HbA1c ≥ 6.5%, the CIs overlapped, which may weaken the strength of the dose–response interpretation. However, this could be attributed to the fact that individuals with diabetes who maintain good glycemic control through medications, diet, and physical activity can achieve HbA1c levels within or close to the normal range. Consequently, the distribution of HbA1c values may not differ substantially between well-controlled diabetic individuals and those without diabetes, potentially attenuating the observed association for this specific outcome [24]. According to current guidelines, individuals who achieve HBsAb levels ≥ 10 mIU/mL within 1–2 months of completing the HBV vaccine series are considered to be protected against HBV infection [25]. However, if achieving higher antibody levels can offer greater protective effects against diabetes, ensuring a higher tier of HBsAb through vaccination might be beneficial.

In the present study, stratified analysis revealed significant geographical differences in the protective effects of HBV immunity against diabetes. Notably, the United States—despite its wealth and advanced healthcare system—showed the least benefit in diabetes prevention associated with HBV immunity. According to the most recent International Diabetes Federation (IDF) Diabetes Atlas 2025, the prevalence of diabetes among adults (20–79 years) varies markedly by region: Africa 4.2%, Middle East and North Africa 17.6%, South and Central America 10.0%, North America and Caribbean 15.1%, Southeast Asia 9.7%, and Western Pacific 12.4% [2]. These marked regional differences in diabetes prevalence may partly explain our findings, highlighting the influence of geographical and population factors in modifying the impact of HBV immunity on diabetes risk. The geographical differences may be influenced by variations in ethnicity, genetic predisposition, dietary habits, and lifestyle factors across regions, all of which are closely linked to diabetes risk. However, due to the limited data on various ethnicities in the TriNetX database, which relies on EMRs, we were unable to conduct further stratified analyses by ethnicity to examine these potential contributors in greater detail.

The age-stratified analysis indicated that the association between HBV immunity and reduced diabetes risk was stronger in younger individuals compared to middle-aged and older individuals. This finding may be attributed to the natural aging of the immune system, also known as immunosenescence, which leads to diminished vaccine-induced immune responses in older adults [26]. A national cohort study by Mereen also demonstrated that responses to the HBV vaccine declined in individuals aged > 40 years and were markedly lower in those aged > 60 years [27]. Additionally, circulating HBsAb levels have been shown to wane over time after vaccination [28]. These factors—waning immunity and immunosenescence—may collectively explain the attenuated protective effect observed in older adults in our study.

The aforementioned phenomenon can be explained as follows. Individuals who develop immunity after vaccination may have stronger immune regulation than others; in other words, they have better immune fitness. Vaccines affect the immune system both directly and indirectly. In addition to protecting against the target disease, vaccines exert indirect effects on other illnesses, contributing to the overall health of vaccinated individuals. One plausible explanation is trained immunity, in which innate immune cells undergo epigenetic reprogramming, leading to enhanced nonspecific responses and improved immune regulation. This mechanism has been demonstrated in response to several vaccines, including BCG and influenza. BCG vaccination, for example, has been linked to reduced all-cause mortality, improved immune responses to other vaccines, and even decreased cancer incidence—effects beyond its protection against tuberculosis. Influenza vaccination has also shown protective associations with reduced cardiovascular events and all-cause mortality, likely via modulation of systemic inflammation [14,15]. Given that chronic inflammation contributes to insulin resistance and type 2 diabetes, HBV vaccination may reduce diabetes risk through similar pathways. These non-specific effects support the potential of HBV immunization not only for hepatitis B prevention but also as a broader strategy for metabolic health.

From a behavioral perspective, individuals who complete vaccination schedules may be more health-conscious and more likely to engage in healthier behaviors, such as maintaining a better diet or exercising regularly. This raises the possibility that health behavior may act as a confounder in the observed association. In addition, comorbid conditions that are closely linked to diabetes—such as hypertension, dyslipidemia, obesity, and liver disease—may also contribute to the association between HBV immunity and diabetes risk. Although we adjusted for these comorbidities and selected lifestyle factors in our propensity score matching, residual confounding cannot be fully excluded.

The potential for the HBV vaccine to prevent both hepatitis B and diabetes mellitus suggests that the HBV vaccine is a unique dual-benefit intervention. Traditional diabetes prevention requires lifestyle changes, dietary adjustments, exercise, or medication, which require long-term commitment and can be costly. By contrast, the HBV vaccine is accessible and cost-effective, especially in regions with a high prevalence of both HBV and diabetes, such as the Asia-Pacific region and Africa [29,30]. Further studies are needed to confirm these effects and investigate the underlying mechanisms. If validated, the HBV vaccine could become a key tool for the prevention of both infectious and chronic diseases.

Our study has several key strengths. First, with a large sample size drawn from a multinational database, the study results can be generalized to different populations, enhancing the reliability and applicability of the results across different populations. Second, PSM was used to reduce the effect of confounding variables. Notably, to the best of our knowledge, this study is the first to explore the dose–response relationship between HBsAb levels and diabetes, potentially leading to some suggestions for future research.

The study has a few limitations. Because the HBV vaccine has been widely administered over the last century and is recommended from early childhood, vaccine history might not be comprehensively recorded in EMRs [25]. Therefore, we did not use vaccine history data. Instead, we relied on serology as an indirect method of interpretation, which may limit the comprehensiveness of our analysis. To define HBV immunization status, we excluded individuals with prior HBV infection by removing those who had had an HBV diagnosis or tested positive for either HBsAg or HBcAb. Then we inferred that HBsAb positivity in the remaining cohort likely reflected vaccine-induced immunity. This classification approach is commonly used in the HBV vaccination guidelines [10]. However, we acknowledge that rare cases of past HBV infection with negative HBcAb results have been reported, primarily due to immunosuppression or the limited sensitivity of serological assays [31,32], which may lead to a small risk of misclassification. In addition, although we applied propensity score matching to minimize confounding, residual confounding may still exist due to unmeasured variables such as health-seeking behavior, vaccination adherence, and detailed socioeconomic status. These factors could influence both HBV immunity and diabetes risk, but were not fully captured in the database. Furthermore, the accuracy of our findings may be affected by the possibility of misdiagnosis, lack of data, and insufficient reporting of certain factors, as is the case with all clinical database studies.

## 5. Conclusions

HBV immunity may be associated with a reduced risk of diabetes, suggesting broader HBV vaccination as a dual-benefit strategy for the prevention of hepatitis B and diabetes, especially in regions with a high prevalence of both diseases. Further studies are needed to confirm these findings and to clarify the mechanisms underlying the role of HBV immunity in diabetes prevention.

## Figures and Tables

**Table 1:** Risk of diabetes mellitus, comparing HBV-immunized group and HBV-unimmunized group (*n* = 291,231).

Outcome	Patients with Outcome		Hazard Ratio ^†^ (95% CI)
	HBV-Immunized Group (HBsAb ≥ 10 mIU/mL)	HBV-Unimmunized Group (HBsAb < 10 mIU/mL)	
Composite outcome ^‡^	27,653	31,209	0.85 (0.84, 0.87)
Diabetes mellitus diagnosis	12,618	13,731	0.89 (0.87, 0.91)
Any anti-hyperglycemic drugs	21,026	24,192	0.84 (0.83, 0.86)
HbA1c ≥ 6.5%	7107	9178	0.75 (0.72, 0.77)

^†^ For outcomes among the HBV-immunized group and the HBV-unimmunized group (after propensity score matching). ^‡^ Composite outcome: ‘Diabetes mellitus diagnosis’ or ‘Any anti-hyperglycemic drugs’ or ‘HbA1c ≥ 6.5%’.

## Data Availability

The original data used in the current study are not available due to the policy restrictions of the TriNetX database.

## Supplementary Materials

The following supporting information can be downloaded at: https://www.mdpi.com/article/10.3390/diagnostics15131610/s1, Figure S1: Dose–Response Effect of Hepatitis B Surface Antibody Levels and Diabetes Mellitus; Table S1: Baseline characteristics of the HBV-immunized group and HBV-unimmunized group before and after propensity score matching; Table S2: Risk of Diabetes Mellitus, comparing HBV-immunized group and HBV-unimmunized group, stratified by region; Table S3: Risk of Diabetes Mellitus, comparing HBV-immunized group and HBV-unimmunized group, stratified by sex; Table S4: Risk of Diabetes Mellitus, comparing HBV-immunized group and HBV-unimmunized group, stratified by age group; Table S5: Risk of Diabetes Mellitus, comparing groups with HBsAb ≥100 vs. <100 mIU/mL and ≥1000 vs. <1000 mIU/mL; Table S6: Definition of variables using the TriNetX Live platform.

## Abbreviations

The following abbreviations are used in this manuscript:

HBV	Hepatitis B virus
HBsAb	Hepatitis B surface antibody
HBsAg	Hepatitis B surface antigen
HBcAb	Hepatitis B core antibody
HR	Hazard ratio
HCOs	healthcare organizations
EMRs	electronic medical records
ICD-10-CM	International Classification of Diseases, Tenth Revision, Clinical Modification
HbA1c	Glycated hemoglobin
CI	Confidence interval
PSM	Propensity score matching
OR	Odds ratio
