# Supplementary material for: Potential Protective Effect of Hepatitis B Immunity Against Diabetes Mellitus: A Retrospective Propensity-Matched Cohort Study
Source: Diagnostics (Basel). 2025 Jun 25;15(13):1610. doi: 10.3390/diagnostics15131610 (PMC12248427; doi:10.3390/diagnostics15131610)
Supplement: Supplementary file 1 [file diagnostics-15-01610-s001.zip › diagnostics-3664203-supplementary.pdf]

Potential Protective Effect of Hepatitis B Immunity Against Diabetes Mellitus: A Retrospective Propensity-Matched Cohort Study

Supplementary Materials

DOSE-RESPONSE ANALYSIS

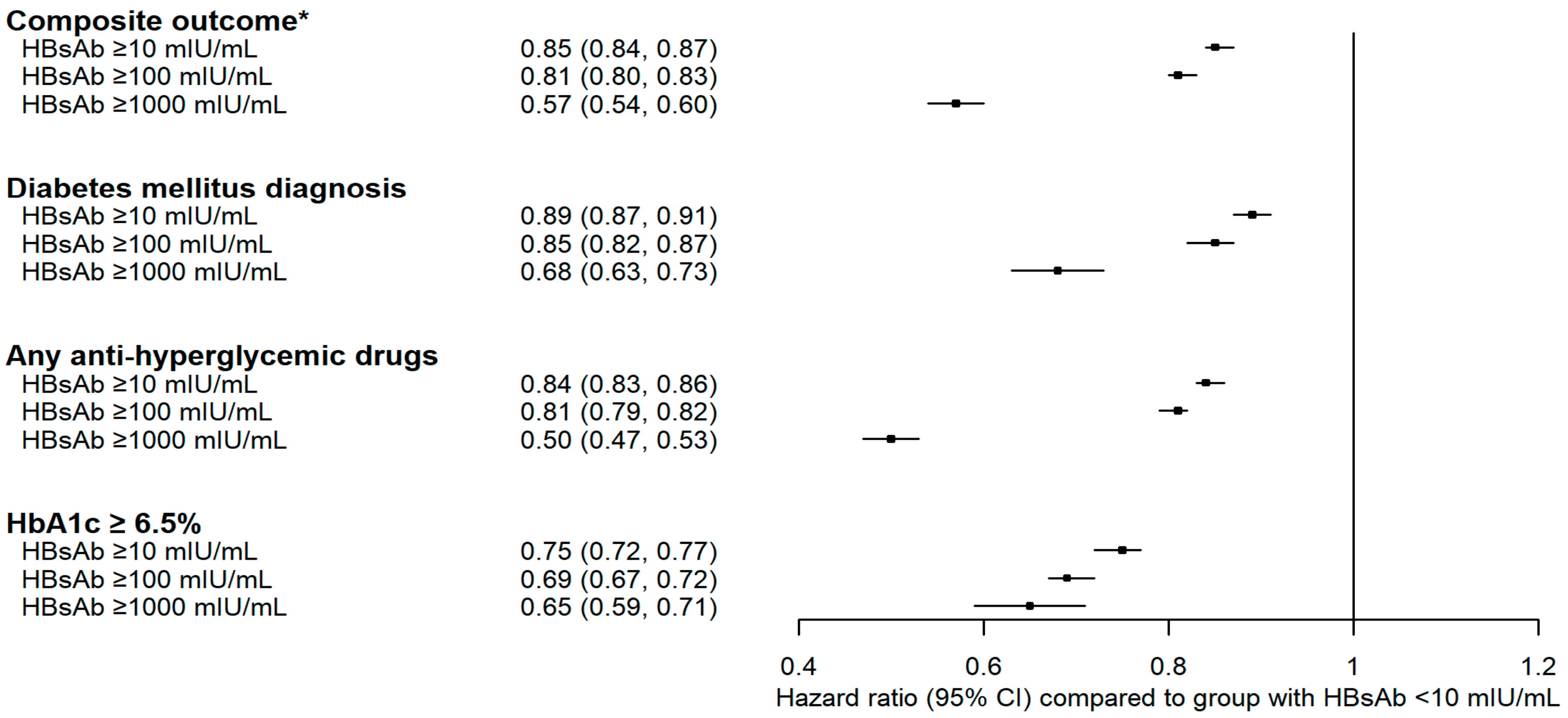

Figure S1: *Dose-Response Effect of Hepatitis B Surface Antibody Levels and Diabetes Mellitus*

*Composite outcome: 'Diabetes mellitus diagnosis' or 'Any anti-hyperglycemic drugs' or 'HbA1c ≥ 6.5%'

Table S1: *Baseline characteristics of the HBV-immunized group and HBV-unimmunized group before and after propensity score matching*

	**Before matching**			**After matching**		
	**HBV-immunized group (HBsAb ≥ 10 mIU/mL) (n = 573,785)**	**HBV-unimmunized group (HBsAb < 10 mIU/mL) (n = 318,684)**	**SMD†**	**HBV-immunized group (HBsAb ≥ 10 mIU/mL) (n = 291,231)**	**HBV-unimmunized group (HBsAb < 10 mIU/mL) (n = 291,231)**	**SMD†**
**Age at index** (Mean +/-SD)	38 +/- 14.5	44 +/- 16.9	0.383	42.1 +/- 16.1	42.1 +/- 16.1	0.002
**Sex**						
Male	235,759 (41.09%)	139,762 (43.86%)	0.056	125,539 (43.11%)	125,836 (43.21%)	0.002
Female	334,541 (58.30%)	174,411 (54.73%)	0.072	162,248 (55.71%)	162,177 (55.69%)	0.000
Unknown Gender	3,485 (0.61%)	4,511 (1.42%)	0.081	3,444 (1.18%)	3,218 (1.11%)	0.007
**Ethnicity**						
Hispanic or Latino	50,154 (8.74%)	31,951 (10.03%)	0.044	30,492 (10.47%)	29,516 (10.14%)	0.011
Not Hispanic or Latino	387,376 (67.51%)	218,547 (68.58%)	0.023	192,384 (66.06%)	197,341 (67.76%)	0.036
Unknown Ethnicity	136,255 (23.75%)	68,186 (21.40%)	0.056	68,355 (23.47%)	64,374 (22.10%)	0.033
**Race**						
White	268,796 (46.85%)	188,870 (59.27%)	0.251	160,235 (55.02%)	166,070 (57.02%)	0.040
Asian	60,939 (10.62%)	11,160 (3.50%)	0.281	11,590 (3.98%)	11,160 (3.83%)	0.008
Black or African American	67,463 (11.76%)	40,921 (12.84%)	0.033	41,355 (14.20%)	38,596 (13.25%)	0.028
American Indian or Alaska Native	2,971 (0.52%)	1,131 (0.36%)	0.025	1,083 (0.37%)	1,120 (0.39%)	0.002
Native Hawaiian or Other Pacific Islander	702 (0.12%)	350 (0.11%)	0.004	348 (0.12%)	341 (0.12%)	0.001
Other race	25,555 (4.45%)	12,122 (3.80%)	0.033	12,265 (4.21%)	11,859 (4.07%)	0.007
Unknown Race	147,359 (25.68%)	64,130 (20.12%)	0.133	64,355 (22.10%)	62,085 (21.32%)	0.019
**BMI** (Mean +/- SD)	27.18 +/- 6.52	28.79 +/- 7.21	0.234	27.78 +/- 6.68	28.72 +/- 7.26	0.136
**HbA1c** (Mean +/- SD)	5.32 +/- 0.53	5.41 +/- 0.52	0.168	5.35 +/- 0.54	5.39 +/- 0.52	0.089

**Socioeconomic and psychosocial circumstances**	16,210 (2.83%)	10,358 (3.25%)	0.025	9,424 (3.24%)	9,417 (3.23%)	<0.001
**Lifestyle**						
Tobacco use	11,897 (2.07%)	11,541 (3.62%)	0.093	8,899 (3.06%)	8,854 (3.04%)	0.001
Nicotine dependence	41,573 (7.25%)	35,536 (11.15%)	0.135	29,477 (10.12%)	29,340 (10.07%)	0.002
Alcohol-related disorders	21,315 (3.72%)	17,598 (5.52%)	0.086	14,413 (4.95%)	14,708 (5.05%)	0.005
**Comorbidities**						
Hypertensive diseases	67,552 (11.77%)	59,204 (18.58%)	0.191	46,667 (16.02%)	46,002 (15.8%)	0.006
Hyperlipidemia	50,742 (8.84%)	45,164 (14.17%)	0.168	35,055 (12.04%)	34,697 (11.91%)	0.004
Heart failure	8,501 (1.48%)	8,843 (2.78%)	0.090	6,218 (2.14%)	6,140 (2.11%)	0.002
Ischemic heart diseases	14,926 (2.60%)	15,178 (4.76%)	0.115	10,728 (3.68%)	10,427 (3.58%)	0.006
Chronic kidney disease (CKD)	26,763 (4.66%)	14,322 (4.49%)	0.008	12,903 (4.43%)	13,453 (4.62%)	0.009
Overweight and obesity	40,884 (7.13%)	34,600 (10.86%)	0.131	29,241 (10.04%)	28,297 (9.72%)	0.011
Liver diseases	33,691 (5.87%)	31,132 (9.77%)	0.146	24,406 (8.38%)	23,750 (8.16%)	0.008

†SMD: standardized mean difference.

STRATIFIED ANALYSIS BY DEMOGRAPHIC VARIABLES

Table S2: *Risk of Diabetes Mellitus, comparing HBV-immunized group and HBV-unimmunized group, stratified by region*

**Outcome**	**Hazard ratio (CI 95%)**				
	**Global Network**	**US**	**LATAM**	**EAMA**	**APAC**
Composite outcome**	0.85 (0.84, 0.87)	0.90 (0.89, 0.92)	0.64 (0.60, 0.68)	0.53 (0.48, 0.58)	0.73 (0.65, 0.83)
Diabetes mellitus diagnosis	0.89 (0.87, 0.91)	0.91 (0.89, 0.94)	0.93 (0.77, 1.11)	0.49 (0.42, 0.56)	0.74 (0.64, 0.85)
Any anti-hyperglycemic drugs	0.84 (0.83, 0.86)	0.90 (0.89, 0.92)	0.57 (0.53, 0.61)	0.48 (0.43, 0.55)	0.71 (0.60, 0.83)
HbA1c ≥ 6.5%	0.75 (0.72, 0.77)	0.75 (0.73, 0.78)	0.81 (0.71, 0.92)	0.60 (0.47, 0.78)	0.77 (0.65, 0.91)

Table S3: *Risk of Diabetes Mellitus, comparing HBV-immunized group and HBV-unimmunized group, stratified by sex*

**Outcome**	**Hazard ratio (CI 95%)**	
	**Male**	**Female**
Composite outcome**	0.87 (0.85, 0.89)	0.84 (0.83, 0.86)
Diabetes mellitus diagnosis	0.91 (0.88, 0.94)	0.87 (0.84, 0.90)
Any anti-hyperglycemic drugs	0.85 (0.83, 0.87)	0.84 (0.82, 0.86)
HbA1c ≥ 6.5%	0.77 (0.74, 0.81)	0.72 (0.69, 0.75)

Table S4: *Risk of Diabetes Mellitus, comparing HBV-immunized group and HBV-unimmunized group, stratified by age group*

**Outcome**	**Hazard ratio (CI 95%)**		
	**Aged 18 to 44**	**Aged 45 to 64**	**Aged 65 and older**
Composite outcome**	0.80 (0.78, 0.82)	0.89 (0.87, 0.92)	0.88 (0.84, 0.91)
Diabetes mellitus diagnosis	0.76 (0.73, 0.79)	0.93 (0.90, 0.96)	0.97 (0.92, 1.03)
Any anti-hyperglycemic drugs	0.79 (0.77, 0.82)	0.89 (0.87, 0.91)	0.85 (0.81, 0.89)
HbA1c ≥ 6.5%	0.62 (0.59, 0.65)	0.79 (0.76, 0.83)	0.79 (0.72, 0.86)

SENSITIVITY ANALYSIS

Table S5: *Risk of Diabetes Mellitus, comparing groups with HBsAb ≥100 vs <100 mIU/mL and ≥1000 vs <1000 mIU/mL*

**Outcome**	**Hazard ratio (CI 95%)**	
	**HBsAb ≥ 100mIU/mL vs HBsAb < 100mIU/mL**	**HBsAb ≥ 1000mIU/mL vs HBsAb < 1000mIU/mL**
Composite outcome**	0.88 (0.87, 0.90)	0.71 (0.68, 0.74)
Diabetes mellitus diagnosis	0.87 (0.85, 0.90)	0.78 (0.73, 0.83)
Any anti-hyperglycemic drugs	0.88 (0.87, 0.90)	0.65 (0.61, 0.68)
HbA1c ≥ 6.5%	0.81 (0.79, 0.84)	0.86 (0.79, 0.93)

CODES USED ON TRINETX

Table S6: *Definition of variables using the TriNetX Live platform*

Variables	Codes
**Codes for inclusion and exclusion criteria**	
Hepatitis B diagnosis (ICD code)	ICD-10: B16, B17.0, B18.0, B18.1, B19.1<br>ICD-9: 070.2, 070.3
HBsAg positive	LOINC: 5195-3, 5196-1, 7905-3, 47364-5, 50967-9, 51659-1, 65633-0, 75410-1
HBcAb positive	LOINC: 13952-7, 16933-4, 24113-3, 31204-1, 32685-0, 47358-7, 47440-3, 51658-3, 51914-0, 83100-8
HBsAb	TNX: 9059
Diabetes mellitus	ICD-10: E08-E13
Any anti-hyperglycemic drugs	ATC: A10A, A10BA, A10BB, A10BF, A10BG, A10BH, A10BK, A10BX<br>(A10 but not included A10BJ "Glucagon-like peptide-1 (GLP-1) analogues")
HbA1c	TNX: 9037
Death	Deceased
**Codes for covariates**	
Age at index date	AI
Gender	
Male	M
Female	F
Unknown Gender	UN
Ethnicity	
Hispanic or Latino	2135-2
Not Hispanic or Latino	2186-5
Unknown Ethnicity	UN
Race	
White	2106-3
Asian	2028-9
Black or African American	2054-5
American Indian or Alaska Native	1002-5
Native Hawaiian or Other Pacific Islander	2076-8
Other Race	2131-1
Unknown Race	UNK
BMI	9083
Socioeconomic and psychosocial circumstances	Z55-Z65

Lifestyle	
Tobacco use	Z72.0
Nicotine dependence	F17
Alcohol-related disorders	F10
Comorbidities	
Hypertensive diseases	I10-I1A
Hyperlipidemia	E78
Heart failure	I50
Ischemic heart diseases	I20-I25
Chronic kidney disease (CKD)	N18
Overweight and obesity	E66
Liver diseases	K70-K77
**Codes for outcomes**	
Diabetes mellitus	E08-E13
Any anti-hyperglycemic drugs	ATC: A10A, A10BA, A10BB, A10BF, A10BG, A10BH, A10BK, A10BX<br>(A10 but not included A10BJ "Glucagon-like peptide-1 (GLP-1) analogues")
HbA1c	TNX: 9037
